# Lower Gastrointestinal Bleeding Secondary to Appendiceal Mucinous Neoplasm: A Report of Two Cases and a Review of the Literature

**DOI:** 10.7759/cureus.52908

**Published:** 2024-01-25

**Authors:** Jesús Omar Soto Llanes, Samanta Kin Dosal Limón, Ana Jimena Iberri Jaime, Mario Zambrano Lara, Billy Jiménez Bobadilla

**Affiliations:** 1 General Surgery, General Hospital of Mexico Dr. Eduardo Liceaga, Mexico City, MEX; 2 General Medicine, General Hospital of Mexico Dr. Eduardo Liceaga, Mexico City, MEX; 3 Colorectal Surgery, General Hospital of Mexico Dr. Eduardo Liceaga, Mexico City, MEX

**Keywords:** right hemicolectomy, appendectomy, pseudomyxoma peritonei, lower gastrointestinal bleeding, appendicular mucinous neoplasms

## Abstract

Appendicular mucinous neoplasms, constituting less than 1% of gastrointestinal tract neoplasms, are heterogeneous entities. They may be asymptomatic, discovered incidentally, or present as large tumors due to mucin accumulation. The lack of standardized treatment complicates management. Imaging studies, particularly CT scans, are crucial for diagnosis and follow-up.

This case report presents two clinical cases of women in their sixth and seventh decades of life with a history of lower gastrointestinal bleeding, mild anemia in laboratory studies, and incomplete colonoscopies. The diagnosis, confirmed through CT scans, led to the decision for surgical intervention in both cases, involving laparoscopic right hemicolectomy with ileotransverse anastomosis. Subsequently, histopathological reports confirmed the diagnosis of high-grade appendicular mucinous neoplasms, and a follow-up plan was established with imaging studies every six months with no recurrence at two years.

Over 50% of appendicular tumors are mucinous neoplasms originating from low-grade mucinous neoplasms. Given the low lymph node invasion (2%), appendectomy may suffice if the entire tumor is excised. Extensive resections or right hemicolectomy are reserved for larger tumors or high-grade neoplasms to minimize local recurrence risk. Mucinous neoplasms with acellular mucin and peritoneal invasion may require cytoreduction or right hemicolectomy, while those with mucinous epithelium may need hyperthermic intraperitoneal chemotherapy (HIPEC) due to the risk of local recurrence, worsened by the presence of extra appendiceal epithelial cells. Disease-free and overall survival depend on treatment and initial lesion characterization. A five-year survival rate of 86% is reported for low-grade mucinous neoplasms. Follow-up approaches lack an ideal standard, generally involving physical examinations and imaging studies every six months to one year during the first six years.

## Introduction

Appendicular mucinous neoplasms represent less than 1% of gastrointestinal tract neoplasms. They are heterogeneous entities with variable presentations that, depending on the stage at which they are found, can either be asymptomatic and discovered incidentally or debut as large tumors due to the accumulation of mucin in the abdominal cavity [1].

The clinical presentation of appendicular neoplasms is often nonspecific, and they are frequently diagnosed at advanced stages when the tumors reach considerable sizes and become evident during physical examination, secondary to mucin accumulation. A presentation resembling acute appendicitis is common, and it is often an incidental diagnosis in appendectomy specimens. However, they can also manifest as abdominal pain, weight loss, and anemia [1, 2].

The classification of appendicular adenocarcinomas is controversial and includes mucinous adenocarcinomas, non-mucinous adenocarcinomas, and ex-goblet-cell-carcinoid adenocarcinomas. The presence of mucin beyond the muscularis mucosae is considered evidence of appendicular malignancy. Subsequently, Misdraji et al. coined the term low-grade appendicular mucinous neoplasm, replacing the term mucocele, and based it on mucin differentiation and grade. High-grade mucinous neoplasms are usually rare, presenting histopathological features of poor differentiation but with an absence of invasion. However, if they exhibit peritoneal invasion, they should be classified as adenocarcinomas due to the risk of peritoneal invasion they pose [1, 3-5].

Imaging studies, especially CT scans, play a crucial role in the diagnosis and monitoring of appendiceal mucinous neoplasms due to their accessibility and affordability. The sensitivity of CT varies depending on lesion size, reaching up to 94% for lesions larger than 5 cm and dropping to less than 20% for lesions smaller than 1 cm [6]. Ultrasonography may be used initially, typically revealing heterogeneous cystic lesions with characteristic signs favoring their diagnosis. However, in most cases, CT is indispensable for a comprehensive diagnostic approach. Magnetic resonance imaging is reserved for specific situations where CT is contraindicated [6,7].

Due to the low lymph node invasion observed in these neoplasms (2%), appendectomy may be considered if the entire tumor is excised. Extensive resections or right hemicolectomy are reserved for tumors larger than 2 cm, involving the periappendiceal area, high-grade neoplasms, or those with invasion beyond the muscularis mucosae to reduce the risk of local recurrence [8-10]. In mucinous neoplasms with acellular mucin and peritoneal invasion, cytoreduction or right hemicolectomy is usually sufficient with close follow-up. In contrast, lesions with mucinous epithelium often require hyperthermic intraperitoneal chemotherapy (HIPEC) due to the risk of local recurrence; however, the presence of extra appendiceal epithelial cells worsens the prognosis [3].

## Case presentation

Case one

A 56-year-old female patient, with a history of type 2 diabetes for 16 years under treatment with metformin 850 mg every 12 hours, presented to the emergency department due to a seven-day history of lower gastrointestinal bleeding. The bleeding was characterized by the presence of hematochezia alternating with rectal bleeding five to six times a day, with an exacerbation of symptoms in the last 48 hours. Upon arrival at the emergency department, the patient had a mean arterial pressure of 60-63 mmHg and a heart rate of up to 108 beats per minute, leading to the decision for admission. Physical examination revealed paleness of the skin and mucous membranes, a hyperdynamic precordium without added phenomena, an abdomen without palpable masses, and an anorectal examination showing hemorrhoidal packages without evidence of bleeding but with evidence of hematochezia on the examining glove. Laboratory tests revealed an elevated lactate level of 4.2 and a decrease in hemoglobin to 8.6 grams per deciliter (Table 1).

**Table 1:** Laboratory results on admission

Analyte	Case 1	Case 2	Normal range
Leucocytes	7.8	4.3	4.5-10 x 10^3^/µL
Neutrophils	65	48	40-70%
Hemoglobin	8.6	9.2	12-16 g/dL
Platelets	235	367	150-450 x 10^3^/µL
Glucose	110	89	74-106 mg/dL
Urea	24	36	17-43 mg/dL
Creatinine	0.89	1.01	0.66-1.09 mg/dL
Sodium	146	137	136-145 mEq/L
Potassium	4.1	3.9	3.5-5 mEq/L
Chloride	101	99	98-107 mEq/L
Magnesium	2.3	2.1	1.9-2.5 mg/dL
Albumin	4.5	3.7	3.5-5.2 g/dL
Carcinoembryonic antigen	4.6	2.2	0-3 ng/mL

**Figure 1:**
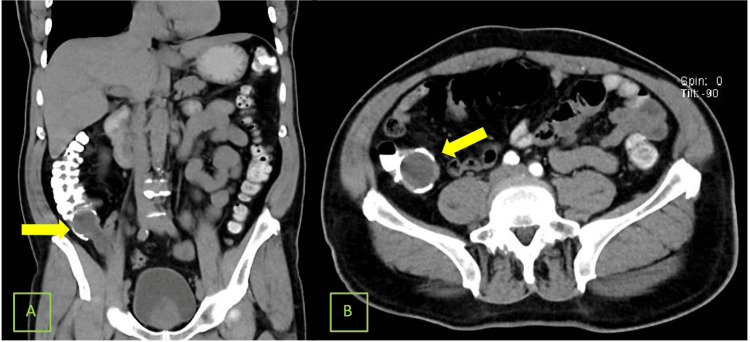
Computed tomography scan (A) Coronal section: The yellow arrow indicates at the base of the appendix an image with a soft tissue density of 25 Hounsfield units (HU). However, it is in close contact with some loops of the distal ileum, and its proximal portion is immersed in the cecum. This lesion has regular, well-defined borders. (B) Axial section: A neoplasm with dimensions of 11.4 x 3.7 x 3.45 cm does not show enhancement after the administration of the contrast medium without demonstrating streaking of adjacent fat or loss of the fat plane.

A colonoscopy was attempted but could not be completed due to the presence of clots in the right colon. Suspecting a possible neoplasm and not finding diverticular disease, a CT was performed, revealing a lesion at the base of the appendix that projects into the cecum, well-defined, homogeneous, with calcified walls, suggestive of appendicular mucinous neoplasm (Figure 1).

**Figure 2:**
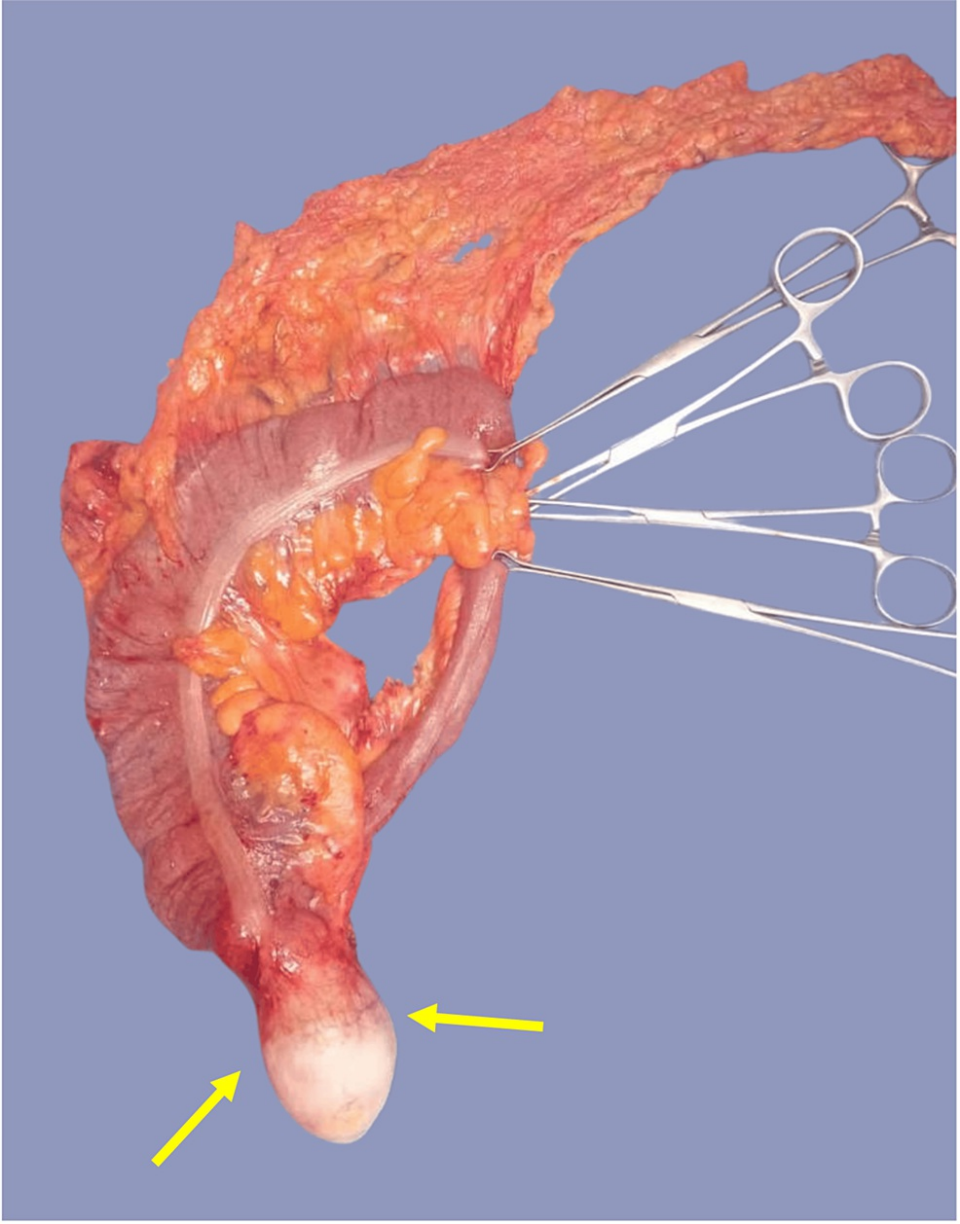
Specimen of right hemicolectomy secondary to mucinous neoplasia of the appendix. Yellow arrows indicate a solid, soft consistency mass with irregularly calcified walls and a slight extension into the cecum, measuring 6.5 x 5.3 x 5 cm.

The complete study protocol, including a chest CT without evidence of distant activity and a carcinoembryonic antigen of 4.6, led to the decision for surgical intervention. A right hemicolectomy with laparoscopic ileotransverse anastomosis was performed without complications (Figure 2).

The patient was started on a liquid diet and progressed well the following day, with adequate tolerance and pain control. Follow-up laboratory tests showed a hemoglobin level of 8.2 grams/deciliter, and the patient was discharged on the fourth day without complications. Follow-up continued through outpatient consultation, where a histopathology report confirmed a high-grade appendicular mucinous neoplasm.

Case two

**Figure 3:**
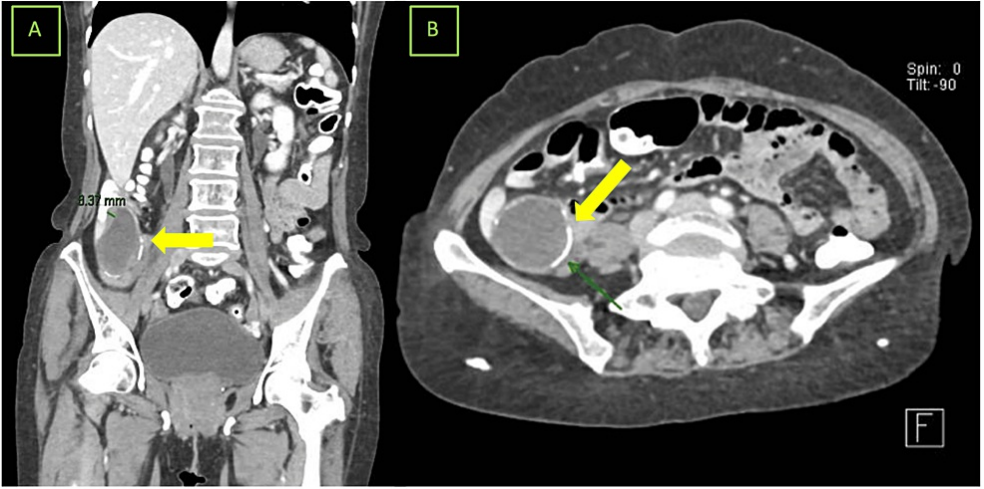
Computed tomography scan (A) Coronal section: The yellow arrow shows a homogeneous cystic-appearing lesion with regular borders and irregular calcification of the walls at the appendicular base, extending to the cecum and terminal ileum without distant activity. (B) Axial section: A mucinous cystic neoplasm at the appendicular base measuring 13.2 x 6.4 x 4.4 cm with regular borders and calcified walls, without evidence of invasion into mesocolonic lymph nodes is seen.

A 63-year-old female patient, with a history of left inguinal hernia repair using mesh 11 years ago and no other significant medical history, came to our outpatient clinic with intermittent episodes of lower gastrointestinal bleeding described as hematochezia persisting for three to four months without additional symptoms. On physical examination, vital signs were within normal parameters, with slight pallor of the skin. The cardiopulmonary examination was unremarkable, and the abdomen was without palpable masses. Anorectal examination revealed hemorrhoidal bundles without abnormalities and no evidence of bleeding. Laboratory tests showed a hemoglobin level of up to 9.2 grams per deciliter (Table 1). A colonoscopy was performed, revealing a subepithelial tumor suggestive of an appendicular mass. A CT scan showed a well-defined, homogeneous lesion with calcified walls, dependent on the appendix, consistent with appendicular mucinous neoplasia (Figure 3).

**Figure 4:**
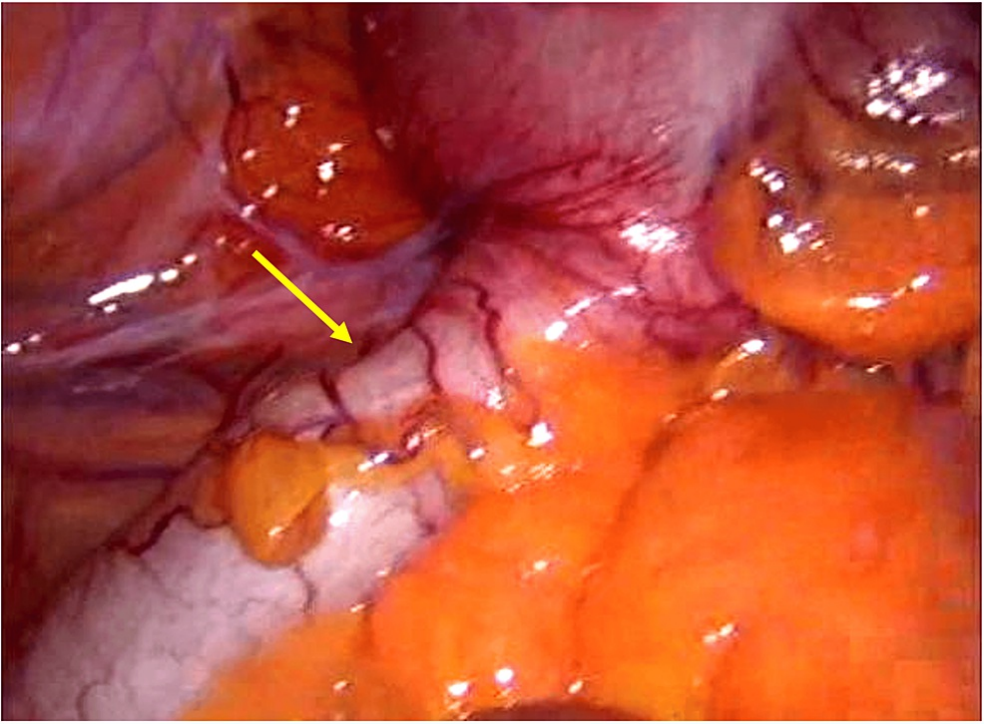
Intraoperative findings The yellow arrow points to an appendicular mucinous neoplasm with a firm consistency, not adhered to deep planes, and without invasion of other structures.

The carcinoembryonic antigen was reported as 2.2. The patient underwent surgical intervention with a laparoscopic right hemicolectomy and ileotransverse anastomosis without complications (Figure 4).

With satisfactory postoperative progress, she was discharged on the fifth day. Follow-up in the outpatient consultation revealed a histopathology report confirming a high-grade appendicular mucinous neoplasm. An imaging follow-up every six months is planned to monitor her condition.

## Discussion

Appendicular mucinous neoplasms account for 0.4%-1% of all gastrointestinal tract neoplasms, with an incidence of 1.2 cases per 100,000 inhabitants per year. There are no significant differences observed between gender and race, with an average age of presentation of 60 years [3].

The clinical course of appendicular mucinous neoplasms will depend on the stage at which they are diagnosed and the histological differences among them. A well-differentiated neoplasm will have a better prognosis than a poorly differentiated one [1]. Mucinous neoplasms are typically discovered incidentally in imaging studies, and most cases present as acute appendicitis (50%). Chronic abdominal pain in the right iliac fossa is another common presentation, and up to 30% of cases may be asymptomatic. Occasionally, they may present as gynecological issues due to suspected ovarian neoplasms. Therefore, imaging studies should be carefully reviewed in these patients to rule out the presence of appendicular neoplasms [4-6].

Imaging studies, especially CT, are the most useful diagnostic tool for the initial assessment of appendicular mucinous neoplasms and are valuable for postoperative follow-up due to their accessibility and cost-effectiveness. However, the identification of peritoneal invasion varies significantly and depends on the size of the lesions. A CT can achieve a sensitivity of up to 94% for lesions larger than 5 cm but decreases to 19%-28% for lesions smaller than 1 cm [6, 10]. An ultrasound may be an initial study in cases of tumor masses or right lower quadrant pain, often necessitating the need for a CT scan. Common ultrasound findings include a heterogeneous cystic lesion in the appendix with variable echogenicity, characteristic of mucinous lesions. The presence of onion skin signs, appearing as laminar images with variable echogenicity due to mucin, typically supports the diagnosis of appendicular mucinous neoplasms [10]. Well-defined masses limited to the appendix or in the lower right quadrant with low attenuation, smooth, thin walls, and curvilinear calcifications resulting from limited inflammatory processes suggest the diagnosis of appendicular mucinous neoplasms, as does an appendix diameter greater than 15 mm [10]. Some preoperative CT classifications, such as the Simplified Preoperative Assessment for Appendix Tumor (SPAAT) score, can predict the possibility of complete cytoreduction with an accuracy of up to 97% [6]. Magnetic resonance imaging does not demonstrate superior effectiveness in showing mucinous lesions compared to CT and is generally reserved for patients who cannot undergo CT. An MRI may be considered when there is doubt about neoplastic rupture, to evaluate peritoneal implants, or to distinguish ovarian cystic lesions [6]. The utility of positron emission tomography and computed tomography (PET-CT) scans is still under study and has limited value in mucinous neoplasms, although it may predict the grade of these lesions in some cases [6]. Colonoscopy is recommended in patients with appendicular mucinous neoplasms to rule out synchronous colorectal tumors, although their incidence is lower compared to primary colorectal neoplasms [6-8]. The role of diagnostic laparoscopy is primarily to identify the resectability of lesions, perform biopsies, and avoid unnecessary laparotomies in late stages that often require more aggressive management with the need for chemotherapy. It is worth noting that exploring the entire abdominal cavity and retroperitoneum may not be initially recommended and should be performed in selected cases [6].

The majority of appendicular adenocarcinomas are mucinous neoplasms in over 50% of cases, originating from low-grade mucinous neoplasms. Initially, the classification suggested by Ronnett for appendicular neoplasms included three variants: disseminated peritoneal adenomucinosis, mucinous peritoneal carcinomatosis, and a third entity that did not correspond to either of the former or had discordant characteristics [1]. Currently, the same authors have simplified this classification by categorizing mucinous neoplasms as high-grade and low-grade [3, 11]. Pseudomyxoma peritonei refers to the presence of diffuse collections in the abdomen or pelvis, as well as implants on the peritoneum, so it is recommended to refer to this entity when the presence of mucin in the abdominal cavity is demonstrated rather than as a histopathological parameter [1, 6].

Low-grade mucinous appendicular neoplasms typically manifest as low-grade proliferation of the mucinous epithelium with obliteration or loss of the lamina propria and muscularis mucosae. Without these characteristics, they would be categorized as adenomas. If they extend beyond the muscularis mucosae or into the subserosa, they are classified as T3, provided they do not involve the visceral peritoneum, regardless of whether it is mucinous epithelium or acellular mucin [4]. Occasionally, perforations are identified in histopathological specimens that are not identified intraoperatively, without evidence of mucin penetrating the muscularis mucosae. Despite not altering the stage, such cases have a higher risk of local recurrence, up to 17% [4]. In instances where positive margins are reported in appendectomies, cytoreductive surgery such as cecectomy or right hemicolectomy may be necessary [4].

It is important to note that Tis in low-grade mucinous neoplasms refers to lesions confined to the muscularis mucosae. The alteration of mucin in the appendicular architecture and the presence of a pseudodiverticulum that pushes the appendicular wall often complicate lesion characterization. Consequently, they cannot frequently be categorized as pT1 and pT2 lesions [3]. The presence of mucinous epithelium extending through the muscularis mucosae and invading the subserosa, or mesoappendix carries a higher risk of tumor recurrence compared to other parts of the digestive tract. Therefore, it should be considered in classification and management decisions [3].

The utility of tumor markers in colorectal neoplasms is well known; however, in appendicular mucinous neoplasms, they remain of limited usefulness. Elevated levels of carcinoembryonic antigen and carbohydrate antigen (CA) 19-9 have been associated in some studies with a decreased likelihood of achieving complete cytoreductions, as well as a decrease in overall survival and disease-free survival. The presence of normal tumor markers in over 70% of patients hinders their use, and sometimes they are only useful in assessing the need for HIPEC [6, 9].

Appendicular mucinous neoplasms exhibit immunohistochemical markers similar to common colorectal neoplasms, being positive for CK20 (100%) and negative for CK7 (71%), along with positivity for MUC5AC (86%) and DPC4 (100%). This suggests a similar pathway to colorectal cancer development, involving the mutation of the KRAS proto-oncogene and the deletion of p53 on chromosome 17. However, the possibility of other pathways being involved in the progression of these neoplasms, such as microsatellite instability, cannot be ruled out [1, 9].

The presence of a low-grade mucinous neoplasm as an incidental finding in an appendectomy is not uncommon. In cases where there are no postoperative findings of residual disease, appendectomy is usually sufficient as treatment for the condition. The need for additional treatment such as cytoreduction or HIPEC should be individualized and depends on the invasion of the visceral peritoneum, lesion size, and lesion grade [6]. Studies have shown that in localized tumors to the appendix, appendectomy is often sufficient as an oncological resection when compared to wider resections, with no impact on five-year overall survival or disease-free survival. The only indication for a more extensive resection is the presence of positive margins in the specimen; however, its management remains controversial [8]. It is important to note that, due to the low incidence of lymph node metastasis, extensive resection is sometimes excessive. In most cases with suspected appendicular mucinous neoplasms, appendectomy is recommended, and in the worst-case scenario, cecectomy or ileocecectomy is performed to avoid the morbidity of a right hemicolectomy. This is usually sufficient for low-grade mucinous neoplasms, where the histopathological report will guide subsequent therapeutic decisions, with the need for right hemicolectomy in 20% of cases due to reports of high-grade neoplasms [12-15].

Perforation or rupture of mucinous neoplasms is the only demonstrated predictive factor favoring progression to pseudomyxoma peritonei, whether it is a primary perforation or iatrogenic during surgery. Therefore, caution should be exercised during the intraoperative period and when reviewing imaging studies suggesting the presence of perforation in the specimen, which may require more aggressive treatment to prevent recurrence and improve disease-free survival [12, 15].

The efficacy of adjuvant chemotherapy has not been demonstrated, and there are no randomized studies available to evaluate it. The decision to administer adjuvant chemotherapy should be individualized based on each patient's characteristics, with recommendations for poorly differentiated tumors, lymph node invasion, or perforated tumors, with 5-fluorouracil being the most recommended agent [1, 15].

Previously, the treatment of mucinous neoplasms relied on continuous or intermittent drainage of ascites, followed by surgeries to achieve maximal tumor cytoreduction, with the eventual need for HIPEC in some cases. The purpose of HIPEC is to provide aggressive local chemotherapy at an elevated temperature (40°C-42°C) to increase the cytotoxicity of chemotherapy agents with minimal systemic effects, aiming to eradicate microscopic disease. Agents such as oxaliplatin (460 mg/m2), mitomycin C (10-12.5 mg/m2), 5-fluorouracil, and cisplatin are used, either individually or in combination [6]. The complete cytoreduction of the disease is a prognostic factor for survival, defined as the absence of visible evidence of disease (CCR0). Sugarbaker developed the intraoperative peritoneal cancer index with a range of 1-39, setting the cutoff point at 20 as an indicator of a low probability of achieving complete cytoreduction [1].

Complete cytoreduction with HIPEC can lead to a survival rate of up to 86%, decreasing to 20% when complete cytoreduction is not achieved. The use of neoadjuvant chemotherapy has not demonstrated effectiveness in overall survival or disease-free survival and is associated with worse outcomes. Therefore, it is not considered useful in these conditions at this time [1].

Disease-free survival and overall survival depend on the treatment and initial characterization of the lesions, with a five-year survival rate for low-grade mucinous neoplasms of 86% [16, 17]. There is no ideal standard for the follow-up of appendicular mucinous neoplasms, and it is generally individualized. The most recent recommendations suggest physical examination every six months, along with imaging studies every six months to one year during the first six years [6, 18-20].

## Conclusions

Appendiceal mucinous neoplasms are rare entities, accounting for less than 1% of digestive system neoplasms, and present in various forms depending on the diagnosed stage. They may be incidentally discovered in imaging studies, histopathological reports of appendectomies, or as large abdominal masses related to mucin accumulation.

The variability in classification systems complicates establishing a standardized treatment approach, thereby worsening the prognosis in advanced stages. The utility of tumor markers remains debated; however, patients with elevated carcinoembryonic antigen levels often experience a poorer long-term prognosis.

Computed tomography remains the gold standard for approaching these neoplasms, though ultrasound can be useful in their initial assessment. Treatment depends on stage, size, and histopathological grade, with appendectomy often sufficient for early stages, while more extensive resections, cytoreductions, and HIPEC may be necessary for advanced stages or those with poor prognostic factors.
